# Navigating Curriculum Changes: Evidence‐Based Literature for Nurse Educators

**DOI:** 10.1155/nrp/7609893

**Published:** 2026-04-22

**Authors:** Nomawabo Lessie Luzipo, Khumoetsile Daphney Shopo, Richelle Van Waltsleven

**Affiliations:** ^1^ School of Nursing, NuMIQ Focus Area, North-West University, 11 Hoffman Street, Potchefstroom, 2520, South Africa, nwu.ac.za

**Keywords:** curriculum change, curriculum implementation, curriculum innovation, curriculum reform, curriculum transition, nurse educators, nursing education, public nursing education institution

## Abstract

**Background:**

Innovation in nursing education curricula is essential to support the evolving challenges of contemporary nursing practice and the difficulties of healthcare systems. However, transitioning to a new curriculum presents significant challenges for nurse educators, who must rapidly adjust to content revision, teaching methods, and assessment strategies, often in the context of limited institutional support.

**Aim:**

To identify the best available evidence for nurse educators transitioning from one curriculum to another in nursing education institutions (NEIs).

**Methods:**

Whittemore and Knafl’s five‐stage framework was used to conduct an integrative literature review: problem identification, literature search, data evaluation, data synthesis, and data presentation. A structured search was performed across CINAHL, EBSCOhost, Google Scholar, Medline, and Scopus. Eligible studies were published in English between 2010 and 2024, available in full text. No geographical limitations were applied to ensure a global perspective. Studies published before 2010 and those not aligned with the research objective were excluded. The selection process was reported in accordance with the preferred reporting items for systematic reviews and meta‐syntheses guidelines, and Covidence software was used to facilitate systematic screening and appraisal, following the critical appraisal skills program guidelines.

**Results:**

Analysis of 22 articles yielded five themes: leadership influence and change management; lack of quality assurance in NEIs; inadequate resources to implement a new curriculum; stakeholder engagement, ownership, and collaboration; and faculty capacity development and training needs for curriculum implementation.

**Conclusion:**

This review concludes that meaningful and sustainable curriculum change in NEIs demands a whole‐system, strategically led approach characterized by inclusive leadership, institutional readiness, coordinated resourcing, faculty development, and stakeholder engagement to overcome historical challenges and effectively prepare competent, adaptable graduates for the complexities of 21st‐century healthcare.

## 1. Introduction

According to the World Health Organization (WHO), innovation in nursing education curricula is crucial for meeting the evolving demands of modern nursing practice and addressing the complexities of contemporary healthcare environments [1, 2]. The growing need to deliver high‐quality, person‐centered care requires a transformative approach to nursing education that equips graduates with the ability to think critically, adapt to change, and function effectively across diverse clinical settings [3]. From an organizational change and educational reform perspective, curriculum transformation is a dynamic and iterative process that requires sustained structural, cultural, and leadership alignment to achieve meaningful institutionalization. The long‐term success of curriculum reform extends beyond initial implementation and requires deliberate strategies to ensure sustainability and institutional integration. Sustaining transitions to a concept‐based education (CBE) curriculum requires intentional leadership, ongoing faculty development, structured evaluation processes, and continuous stakeholder engagement to ensure long‐term integration and effectiveness beyond initial implementation [4–6]. The nursing education changes frequently involve transformation from traditional, content‐driven curricula to modern frameworks that foster holistic nursing competencies [7].

Nursing education institutions (NEIs) are formally accredited higher education or training institutions responsible for preparing individuals for professional nursing practice through structured theoretical instruction, clinical training, research, and professional socialization in accordance with regulatory and professional standards [8–10]. Nurse educators are registered professional nurses who are academically and professionally prepared to design, implement, evaluate, and revise nursing curricula; facilitate theoretical and clinical learning; mentor students; and contribute to scholarship and leadership within NEIs [9]. While curriculum innovation is necessary and increasingly widespread, transitioning to a new curriculum presents significant challenges, particularly for nurse educators [11]. Nurse educators are expected to adapt rapidly to new teaching methodologies, assessment strategies, and curricular content, often with limited institutional support [7]. Common barriers to successful curriculum transition [4, 12] include misalignment between curricular competences and healthcare system needs, shortages of qualified teaching staff, gaps in pedagogical preparation, and inadequate teaching and physical resources. Furthermore, poorly managed transitions can negatively affect nurse educator satisfaction, morale, and retention, thereby undermining the quality and sustainability of nursing education [13]. Despite the critical role of educators in facilitating curriculum changes, there is a limited synthesis of evidence on their experiences and the challenges they face during transitions, particularly within NEIs. Therefore, this integrative literature review seeks to identify the best available evidence on how nurse educators in NEIs navigate curriculum transitions, to inform more effective and supportive change strategies.

This integrative literature review intends to address this gap by systematically examining the existing literature on nurse educators’ experiences, challenges, and support needs during curriculum transition processes in NEIs. By identifying evidence‐based best practices and highlighting areas that require further investigation, the review aims to inform institutional strategies and approaches for enhancing support to nurse educators during curriculum transition. Strengthening support mechanisms is crucial for ensuring the successful implementation of the curriculum, promoting educator satisfaction and retention, and ultimately enhancing the quality of nursing education and graduate outcomes.

## 2. Material and Methods

This study aims to identify the best available evidence to support nurse educators in transitioning from one curriculum to another in NEIs. The objective is to critically appraise and synthesize relevant literature to provide evidence‐based strategies and guidelines that can facilitate effective curriculum transition in NEIs.

Accordingly, the guiding research question is: What is the best available evidence for nurse educators on transitioning from one curriculum to another in NEIs? The review question has been structured using the PICO framework: Population = nurse educators; Intervention/issue = curriculum transition; Context = NEIs; Outcome = educator experiences, challenges, and support strategies. To comprehensively address the research question and develop a robust synthesis of the existing literature, this review adopts an integrative literature review approach, enabling the inclusion of diverse forms of evidence.

**FIGURE 1:**
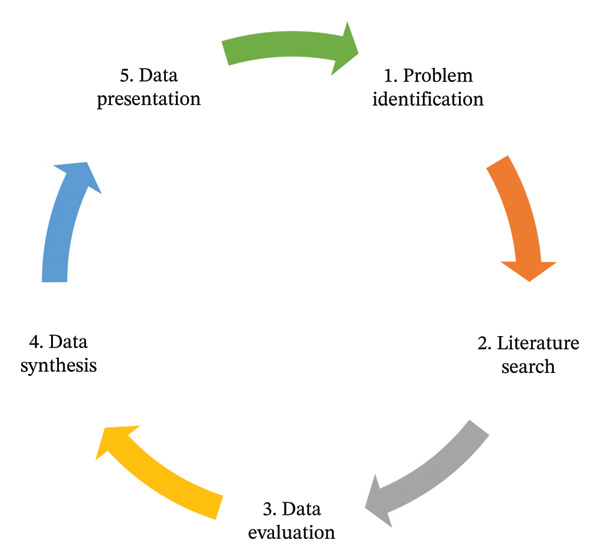
Five‐step framework (Whittemore and Knafl [14]: 547).

This integrative literature review was conducted using the five‐step framework developed by Whittemore and Knafl [14, 15], which is widely recognized for its rigor in synthesizing diverse forms of evidence within nursing and healthcare research [16, 17]. The framework comprises the following steps (see Figure 1): problem identification, literature search, data evaluation, data synthesis, and data presentation [14]. This method was chosen as it accommodates both empirical and theoretical literature [18–20], allowing for a comprehensive understanding of nurse educators’ experiences and challenges during curriculum transitions in NEI. The review was conducted systematically to ensure transparency, reproducibility, credibility, and trustworthiness, and to adhere to the Preferred Reporting Items for Systematic Reviews and Meta‐syntheses (PRISMA) guidelines [21].

The following subsections outline the specific procedures used at each stage of the review process, including the search strategy, selection criteria, data evaluation, and data analysis procedures. An overview of the entire review process is presented in Figure 1, which illustrates the flow diagram of the process from identification to final report.

### 2.1. Step 1: Problem Identification

This step in this review involved identifying a central problem within NEIs, namely, the challenges encountered by nurse educators during the transition from one curriculum to another. In many NEIs, ongoing curriculum changes such as the introduction of competency‐based education (CBE), alignment with national qualifications frameworks, and the incorporation of global nursing standards necessitate that educators adopt new pedagogical approaches and institutional strategies [16]. While such curriculum changes are essential for enhancing the quality and relevance of nursing education, they simultaneously place significant demands on educators. Evidence indicates that nurse educators often struggle to adapt effectively to these transitions [17], primarily due to insufficient institutional support, limited opportunities for professional development, and systematic constraints within educational environments. These challenges can hinder the successful implementation of curriculum innovations and compromise the quality of teaching and learning. Against this backdrop, the present review sought to address the identified gap by systematically examining and synthesizing the best available evidence on the experiences, challenges, and strategies that can support nurse educators in navigating curriculum transitions within NEIs.

### 2.2. Step 2: Literature Search

A comprehensive literature search was conducted to determine the best available evidence for nurse educators transitioning from one curriculum to another in NEIs. This search was guided by the primary research question and objective, which focused on understanding how nurse educators adapt to curriculum transitions within the NEIs. The literature was conducted across multiple databases, including CINAHL, MEDLINE, Scopus, and databases accessed through the EBSCOhost platforms. Google Scholar was used as a supplementary source to capture studies not indexed in these databases. The search strategy was refined with the assistance of a senior university librarian. Search keywords were carefully selected to align with the topic and research objective, including terms such as “*curriculum transition,*” “curriculum change,” “curriculum reform,” and “curriculum innovation” in combination with “curriculum implementation, “nursing education,” “nurse educators,” and references to NEIs. Boolean operators (AND/OR) were effectively used to refine the scope of the search. An initial broad search yielded approximately 22,000 studies. To manage this volume and improve relevance, limiters in the form of inclusion and exclusion criteria were used [22]. This reduced the final pool of literature to 1602 articles, forming the basis for the next stage of rigorous selection.

The inclusion criteria were designed systematically to ensure the relevance and quality of the literature included in the review. Literature published between 2010 and 2024 was reviewed to capture the most current evidence related to curriculum transitions. The review was limited to studies published from 2010 onward, as this period reflects intensified global and national reforms in nursing education, including the shift toward competency‐based curricula, regulatory restructuring, and alignment with contemporary health system priorities [23]. This timeframe ensured the inclusion of evidence relevant to current curriculum transition processes in NEIs. The review was restricted to studies published in English, a language in which the authors are proficient, to ensure accurate interpretation of findings and to avoid additional translation costs. Only studies available in full text were included to enable a comprehensive appraisal of the methodology, findings, and conclusions, thereby ensuring rigorous quality assessment, accurate data extraction, and transparency in the review process [24]. No country limitations were imposed to maintain a global perspective. The included studies demonstrated wide geographic representation, encompassing high, middle, and low‐income countries across North America, Europe, Asia, and Africa, such as the United States, Australia, the United Kingdom, New Zealand, Israel, China, Pakistan, Rwanda, Namibia, Lesotho, and South Africa.

Studies conducted in public, private, or mixed higher education settings, including multiprofessional samples and secondary reviews, were included where findings were transferable to curriculum transitions in nursing education. Curriculum reform is not sector‐bound, as leadership, governance, and sustainability mechanisms often transcend institutional contexts. Broader inclusion enabled the identification of interdisciplinary, theoretically grounded evidence relevant to sustaining curriculum reform in nursing education. Conversely, studies published before 2010 and those not aligned with the research objective were excluded.

**FIGURE 2:**
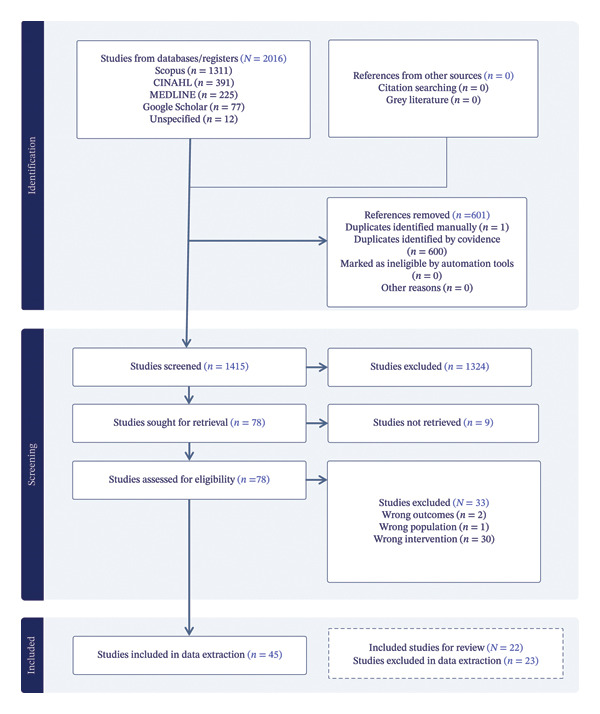
PRISMA flow diagram, Covidence 25 software tool (Page et al. [25]).

To ensure transparency and academic rigor, PRISMA guidelines were used to report the selection process [25]. The adapted PRISMA flow diagram visually depicts each phase of article identification, screening, eligibility, and final inclusion presented by the Covidence tool [26]. The Covidence tool enhanced the integrity of the review by offering a structured approach (see Figure 2) for tracking and reporting the results of the literature selection process [26]. It also confirmed the methodological soundness of the review, increasing its credibility and reliability. Ultimately, this integrative literature review appraises summarized peer‐reviewed literature that offers evidence on the challenges and best practices for transitioning nurse educators to new curricula within NEIs. The final sample of studies reflects a diverse yet focused body of evidence, used to address the research question. This methodological foundation not only guaranteed the validity of the results but also provided a clear framework for synthesizing findings across different educational settings. Figure 2 presents the adapted PRISMA flow diagram, reported by the Covidence 25 software tool [25].

### 2.3. Step 3: Data Evaluation

This section presents a critical evaluation of the literature selection for the integrative review, which was managed using the Covidence 25 software tool [26]. A systematic and transparent approach was adopted to ensure that only high‐quality and relevant studies were retained for synthesis. Each study was assessed against predetermined criteria focused on methodological rigor, relevance to the research question, and the trustworthiness of the reported findings.

Methodological rigor was determined by considering the study design, appropriateness of sampling strategies, and robustness of data collection and analysis methods. Relevance was evaluated by determining the extent to which each study addressed issues related to curriculum transition in NEIs, with particular attention given to the experiences and support needs of nurse educators.

The Covidence tool enabled the management of large volumes of references, facilitated automatic detection of duplicates, and provided a structured process for blinded independent review by two researchers. Blinding was used to minimize selection bias and ensure the integrity of the decision‐making process during title, abstract, and full‐text screening [26]. Initially, a total of 2016 studies were imported into Covidence from databases: CINAHL, EBSCOhost, Medline, Scopus (saved on RIS file), and Google Scholar (saved on EndNote file). Covidence automatically removed 600 duplicates, and 1 additional duplicate was removed manually, resulting in 1415 studies eligible for title and abstract screening. Following this step, 1324 studies were excluded for being irrelevant to the research question, leaving 78 studies for full‐text screening. During the full‐text review, 33 studies were excluded for reasons including wrong intervention (*n* = 30), wrong outcome (*n* = 2), and wrong population (*n* = 1), and 9 studies were not retrieved. A structured data extraction process was undertaken to systematically collect and organize relevant information from the included studies, ensuring consistency, accuracy, and transparency in the analysis (see Table 1). A total of 45 studies were included in the data extraction process and subsequently synthesized. These studies were captured on a standardized tool to facilitate the process.

**TABLE 1:** Sample of data extraction adopted from reference [27].

Author	Population & sample size	Study setting	Study design	Summary of findings	Conclusion
[28]	15 nurse educators	School of Nursing at the Rundu Campus, one of the 12 campuses of the University of Namibia, is located in North‐Eastern Namibia	The study employed an exploratory, descriptive, and contextual qualitative design	This review highlights that curriculum reform in nursing education institutions is a complex, multidimensional process requiring aligned leadership, resources, stakeholder engagement, quality assurance, and faculty development, particularly in public NEIs with entrenched systemic challenges	It concludes that successful curriculum transformation requires strategies including digital skills training, provision of technological resources, strengthened research, and active stakeholder involvement
[29]	Eight nurse educators	Two universities in one Western state, USA	Grounded theory design	Effective curriculum reform depends on sufficient resources, strong leadership, early stakeholder engagement, and institutional and faculty readiness, but is often hindered by time constraints, resistance to change, misconceptions, and limited faculty curriculum expertise	Transitioning to a concept‐based curriculum required a significant mindset shift, which educators achieved through support that enabled them to create practice‐based learning, refine implementation, and recognize the benefits of the new approach
[30]	21 nurse educators	Five nursing education institutions in Lesotho, a small sub‐Saharan African country	A descriptive, qualitative, multiple‐method research approach	Culturally aligned practices like collectivism and stakeholder engagement facilitated curriculum reform, while barriers included hierarchical resistance, educator challenges with CBC development, and institutional reluctance to implement change	The authors present a context‐specific framework to support educators implementing a transformative nursing curriculum to use facilitative, culturally congruent strategies
[31]	10 nurse educators	A public NEI with two campuses in North‐West Province, South Africa, which implemented R171 in 2021	Descriptive phenomenology research design	Nurse educators need to be actively involved in curriculum planning, with strong institutional support, particularly for logistics like transportation, as a lack of resources, limited time, and inadequate support for clinical duties contribute to significant emotional stress	Effective implementation of the R171 nursing curriculum requires the active involvement of nurse educators in its development, along with comprehensive support through adequate essential resources
[32]	19 participants (4 senior academics and 15 academics across professions and specialties involved in the curricula)	This study occurred in a Faculty of Health and Social Care in one HEI in the United Kingdom	A qualitative design using a single holistic case study approach was adopted	The distributive and collective leadership encouraged broad support for curriculum implementation, but academics felt excluded from strategic decisions, with emotional dynamics both hindering innovation at leadership levels and boosting engagement operationally, while top‐down approaches undermined inclusivity and readiness for change	A widespread testing of the new model, including its impact on personal change and well‐being, could be valuable, while the persistent gap in understanding the role of emotion in curriculum, practice, and organizational change highlights the need for further research
[33]	25 participants (1 executive, 3 senior managers, 2 academics, and 19 students)	Faculty of Health and Social Care in an HEI in England and four primary care trusts (PCTs) (now clinical commissioning groups (CCGs))	Qualitative design using a case study approach	The findings indicate that leadership was crucial in initiating the program, but guiding teams primarily managed rather than led the change, resulting in a disconnect where frontline academics felt disempowered despite high stakeholder and student empowerment. In addition, limited short‐term successes, challenges to the change’s credibility, and insufficient understanding of the primary care pathway hindered the program’s full integration into academia	Challenges arose between strategic and operational levels during implementation, revealing that the business change model was unsuitable for nursing curriculum changes, leading to the development of a new change model and supporting tool for future reforms
[34]	54 African countries, midwifery education	South Africa	Scoping review	Successful implementation of competency‐based education (CBE) requires understanding contextual factors, engaging stakeholders by defining their roles and opinions, and developing intervention tools to guide the curriculum reform process	Effective CBE implementation requires concurrent planning for sustainability, with ongoing training and support provided to faculty, institutions, policymakers, professional bodies, students, and other stakeholders
[35]	107 nurse educators	Israeli nursing faculty members in a nursing school or university, or a college nursing department	A quantitative, cross‐sectional correlational design.	The findings highlight that organizational climate, management style, and faculty concerns impact curriculum implementation, emphasizing the need for strong organizational support and administrative leadership to address these concerns and foster successful innovation	A collaborative approach to core curriculum revision that includes educators and provides advanced training on innovation actively involves them and helps alleviate their concerns during implementation
[36]	7 lectures and 1 focus group	Public institutions, private institutions, Māori cultural organizations. University of Auckland, New Zealand	A qualitative research design	The findings show that while the new curriculum was essential to meet New Zealand’s evolving healthcare needs, its development was challenging, requiring strong leadership to foster collaboration and ensure that the rights of the Māori people were upheld in line with the Treaty of Waitangi	The development of the BN curriculum demonstrated the Department’s readiness for innovation to meet diverse student and service needs, with the study highlighting the critical role of collaboration
[37]	Seven papers were involved	South Africa	Rapid review methodology	Curriculum change is hindered by limited training, resources, and emotional resistance, but can be facilitated by faculty development, strong leadership, and early engagement factors, also relevant to implementing emerging content like planetary health	This study identified key enablers for integrating planetary health into the undergraduate nursing curriculum, aiming to equip nurses for practice in climate‐affected healthcare environments
[38]	Seven articles were included in the study	The United States and South Africa	Scoping literature review	Common themes across the models included establishing a shared vision, active leadership involvement, representative committees with regular meetings, clearly defined roles, collaborative identification of key concepts and competencies, and the provision of training and resources to ensure consistent implementation of change	The persistent academic‐practice and experience‐complexity gaps in nursing highlight the need for collaboration between nursing programs and clinical partners, with the updated AACN (2021). Essentials offering a timely opportunity to strengthen curricula and enhance graduate readiness through evidence‐based recommendations
[39]	14 nurse educators	The context of this study is Lesotho	Exploratory descriptive interpretive qualitative approach	The findings reveal that while cultural barriers like hierarchy and patriarchy hinder student‐centered learning, facilitators such as national protocols and collectivist values can support transformative curriculum implementation when educators embrace open, reflective teaching practices	Culture plays a crucial yet complex role in implementing a transformative nursing curriculum, with key barriers including power‐based hierarchies, patriarchal norms, authoritative communication styles, and gender dynamics
[40]	Seventeen staff members, including 6 nurse educators	The University of Rwanda	Qualitative and grounded theory	The reform planning for the CBC involved five key steps: forming a curriculum development team, engaging curriculum experts, conducting a situational analysis, developing the curriculum, and preparing stakeholders	A well‐planned reform to competency‐based curricula leads to successful implementation through the involvement of different stakeholders from the beginning
[12]	13 articles	Rwanda	A meta‐synthesis review of qualitative literature	The findings identified two main themes: the urgent need to shift to a competency‐based curriculum (CBC) due to outdated and ineffective traditional models, and the numerous challenges hindering this shift, including inadequate infrastructure, poor collaboration, limited stakeholder involvement, insufficient faculty training, and resource shortages, which together have resulted in superficial and unsustainable CBC implementation	The successful implementation of CBC is hindered by challenges, including secondary‐level nurse education, hospital‐focused programs neglecting primary healthcare, curriculum mismatches with health system needs, shortages of qualified teaching staff, inappropriate teaching methods, and inadequate physical resources
[41]	21 participants: administrators (*n* = 12), nurse educators (*n* = 5), and clinical instructors (*n* = 4)	Five midwifery education institutions in Lesotho	Qualitative design	Most institutions lacked educational quality assurance processes and student‐centered teaching materials, relied on traditional methods linked to the previous curriculum, had insufficient CBC‐related policies, and faced challenges from staff turnover and limited professional development, all undermining effective CBC implementation	The article argues that monitoring and support are crucial for sustaining curricular innovation in midwifery education, with regulation by professional bodies enhancing accountability at institutional, programmatic, and classroom levels
[42]	Participants (*N* = 71): administrators (*n* = 11), facilitators (*n* = 12), and five focus groups with students (*n* = 48)	Lesotho	A qualitative design.	The study reveals that CBM programs largely continued previous content‐driven practices with minimal adaptation, resulting in poor support and monitoring that could negatively impact students, institutions, and patient care	This article describes a gap analysis of stakeholders in Lesotho’s CBM program, revealing a business‐as‐usual approach with minimal system adaptation, negative influences from the previous curriculum, and poor support and monitoring during implementation
[43]	30 articles	South Africa	Integrative literature review	The review found that dedicated offices, officials, or teams played a critical role in leading, supporting, and monitoring curriculum changes, ensuring continuity, accountability, and coordinated leadership throughout the transition	Faculty development, planning, funding, quality assurance, student involvement, and leadership are key tailored strategies that reinforce and sustain planned curriculum changes and should be adopted to support nursing education in Africa and developing countries
[44]	Eight nurse educators	Lesotho	Exploratory qualitative case study approach	Despite receiving CBC training, educators faced significant challenges such as resource shortages, limited institutional and regulatory support, heavy workloads, and a lack of accountability, risking burnout and undermining curriculum reform efforts in low‐resource settings without targeted support and monitoring	This study questions the sustainability of curriculum innovations in low‐income countries, emphasizing that early adopters require ongoing support to ensure long‐term success beyond short‐term investments
[45]	23 original research articles	The United States of America	Systematic review	Transitioning to a CBC requires organized teamwork, administrative support, faculty expansion, diverse teaching methods, and professional development, while careful curriculum mapping and faculty involvement are essential to avoid redundancy and ensure relevance and buy‐in	Teaching in a CBC fosters interactive learning and better theory‐to‐practice integration, but it requires faculty training and support, while also helping to manage the challenge of covering all required competency areas
[46]	123 nursing faculty members	Public nursing schools of Sindh	The analytic cross‐sectional study	Successful transition to a CBC requires teamwork, administrative and faculty support, training, and adequate resources, yet participants reported significant challenges, including uncooperative administration, lack of infrastructure, training, monitoring, and faculty engagement, which, if unaddressed, severely hinder effective implementation	Challenges faced by faculty must be addressed to ensure effective curriculum implementation, which is essential for achieving positive outcomes in the health sector
[47]	30 teaching staff	Australian regional university	A constructivist case study approach	Participants reported a moderate‐to‐high risk of curriculum drift due to limited understanding of key concepts, unclear roles, poor collaboration, and resource constraints, all of which undermined curriculum alignment, implementation, and integrity	The discrepancy is believed to contribute to curricular drift, where the effectiveness of achieving intended learning outcomes from innovative curricula is undermined by a reversion to outdated or unintended practices
[48]	Ten nurse educators	University in Hubei province, Central China	A descriptive qualitative research study	College leaders supported faculty through psychological encouragement, international learning opportunities, and ongoing training, helping them prepare for concept‐based teaching despite facing student resistance, resource shortages, heavy workloads, and self‐doubt challenges that highlight the importance of early preparation and active engagement to foster faculty empowerment and commitment to curriculum reform	Despite facing student resistance, peer maladjustment, and internal conflict, faculty were supported by administrators through change theory–based training, highlighting the need for effective strategies to minimize resistance and foster smoother reform implementation

A total of 45 articles were included for critical appraisal to assess methodological rigor. After this process, 16 studies were rated as high quality, 6 as good/moderate quality, and 23 as low quality. Ultimately, 23 low‐quality studies were excluded for not meeting the inclusion criteria, mainly due to insufficient methodological detail, limited relevance, and inapplicability of findings. Following reviewer’s consensus, 22 studies were retained for inclusion. Data were captured and evaluated using a standardized appraisal tool, as summarized in Table 2.

**TABLE 2:** Critical appraisal of articles included.

Included studies	Critical appraisal tool	Quality rating
[28]	Critical Appraisal Programme Skills (CAPS 2018) checklist	High quality
[30]	Critical Appraisal Programme Skills (CAPS 2018) checklist	High quality
[29]	Critical Appraisal Programme Skills (CAPS 2018) checklist	Moderate quality
[31]	Critical Appraisal Programme Skills (CAPS 2018) checklist	High quality
[32]	Critical Appraisal Programme Skills (CAPS 2018) checklist	Moderate quality
[28]	Critical Appraisal Programme Skills (CAPS 2018) checklist	High quality
[34]	Critical Appraisal Programme Skills (CAPS 2018) checklist	High quality
[35]	Critical Appraisal Programme Skills (CAPS 2018) checklist	High quality
[36]	Critical Appraisal Programme Skills (CAPS 2018) checklist	High quality
[37]	Critical Appraisal Programme Skills (CAPS 2018) checklist	High quality
[38]	Critical Appraisal Programme Skills (CAPS 2018) checklist	High quality
[39]	Critical Appraisal Programme Skills (CAPS 2018) checklist	High quality
[40]	Critical Appraisal Programme Skills (CAPS 2018) checklist	High quality
[12]	Critical Appraisal Programme Skills (CAPS 2018) checklist	Moderate quality
[41]	Critical Appraisal Programme Skills (CAPS 2018) checklist	High quality
[42]	Critical Appraisal Programme Skills (CAPS 2018) checklist	Moderate quality
[37]	Critical Appraisal Programme Skills (CAPS 2018) checklist	Moderate quality
[44]	Critical Appraisal Programme Skills (CAPS 2018) checklist	High quality
[45]	Critical Appraisal Programme Skills (CAPS 2018) checklist	High quality
[46]	Critical Appraisal Programme Skills (CAPS 2018) checklist	High quality
[47]	Critical Appraisal Programme Skills (CAPS 2018) checklist	Moderate quality
[48]	Critical Appraisal Programme Skills (CAPS 2018) checklist	High quality

Data extraction was conducted independently by two reviewers using Covidence’s data extraction tool, which facilitated consistency and completeness in capturing essential information such as study characteristics, research methods, key findings, limitations, and practical implications. Although three reviewers were allocated to the process, consensus was successfully achieved between the two primary reviewers, and therefore, the involvement of the third reviewer as a mediator was not required [14]. Critical appraisal was a crucial part of the evaluation process. It allowed for the assessment of relevance, methodological quality, and credibility of each included study. As noted by Whittemore and Knafl [14], critical appraisal enabled researchers to determine the strength of the evidence and its applicability to the context under review.

This phase helped identify studies with high methodological rigor and discard those that did not meet the required threshold. To ensure objectivity, both reviewers independently appraised the selected studies. They held multiple consensus meetings to resolve discrepancies in screening decisions and the interpretation of findings. The collaborative review process not only increased the reliability and credibility of the study but also reduced potential reviewer bias, contributing to the overall rigor of the literature review [14]. The Critical Appraisal Skills Programme (CAPS) [49] was employed to evaluate the methodological soundness of the selected studies. The CAPS tool provides a standardized framework for assessing various study designs, population selection, data collection and data analysis methods, validity of results, and relevance to practice [49]. This enabled the reviewers to identify potential bias and limitations in the studies and helped decide on the inclusion based on methodological merit [22].

### 2.4. Step 4: Data Analysis

The data analysis followed the systematic data extraction phase and was conducted independently by two reviewers, with the extraction tool piloted in advance to minimize potential bias. The review employed thematic analysis guided by Whittemore and Knafl’s [14] integrated framework, which enabled the synthesis of findings across diverse study designs, allowing findings from both qualitative and quantitative research to be synthesized into a unified interpretation. By applying this structured approach, the analysis went beyond simple aggregation of results to produce an integrative synthesis that captured the complexity of evidence across the included studies. Data were systematically extracted, compared, and organized, with constant comparison used to identify both patterns and differences that informed the development of synthesized themes. Each study was critically appraised for methodological strengths and limitations, thereby enhancing the credibility of the synthesis (see Table 3). Extracted data were coded, rearranged, and categorized into meaningful clusters, from which main themes and subthemes were generated [14]. This process provided a coherent and integrated evidence base that supports both theoretical insight and practical application.

**TABLE 3:** Thematic synthesis table adopted from reference [27].

Themes	Subthemes	Number	Studies that informed themes
1. Leadership influence and change management	Lack of strategic leadership and management support	12	[28, 30, 32–35, 38, 39, 43, 45, 46, 48]
	Lack of vision and communication		
	Leadership role and ambiguity		
	Exclusion of academies from decision‐making		
	Top‐down strategic leadership		
	Inconsistent leadership across levels		
	Low ownership of change		
	Disengagement from reform processes		
	Leadership and management style		
	Strong administrative vision and support are needed		
	Faculty experience and expectations		
	Mobilizing faculty for innovation		
	Faculty buy‐in is critical for innovation		
	Need for communication and shared vision		
	Organizational climate influences		
	Faculty educational experience influences and readiness		
	Organizational and structured support systems are essential for success		
	Institutionalization of leadership structures		
	Designated leadership roles		
	Ensuring continuity and coordination		
	Risk of burnout and curriculum failure		
	Power dynamics		
2. Lack of quality assurance in nursing education institutions (NEIs)	Lack of collaborative networks for national and international benchmarking	11	[12, 28, 31, 34, 37, 41–43, 45, 46, 48]
	Absence of institutional policies		
	No administration or monitoring policies		
	Monitoring of curriculum change		
	Absence of peer review and supervision		
	Weak internal accountability structures		
	Lack of quality monitoring mechanisms		
	Absence of review and monitoring systems		
	Absence of implementation feedback loops		
	Absence of curriculum implementation policies		
	Risks to student outcomes and consequences of poorly managed change		
	Lack of accountability within the institution		
	Incomplete teaching plans and teaching guides		
	Lack of ensuring continuity and coordination		
	Running a curriculum without monitoring and evaluation systems		
	Reliance on outdated teaching methods		
	Lack of policies for preparation strategies		
3. Inadequate resources to implement a new curriculum	Lack of teaching tools, infrastructure, need for equipping, library, classrooms, and laboratories (simulation and computer)	11	[12, 28, 30, 31, 37, 41, 44–48]
	Inadequate resources and delayed new curriculum rollout		
	Transportation and logistics challenges		
	Resource constraints in clinical teaching		
	Staff shortage and workload challenges		
	Time constraints and curriculum overload		
	Educator workload and role strain		
	Risk of educator burnout		
4. Stakeholder engagement, ownership, and collaboration	Early involvement of stakeholders	11	[28–31, 33, 34, 36, 40, 44, 47, 48]
	Stakeholder identification and engagement		
	Participatory planning, early consultations, and inclusive dialogs		
	Strategic planning for curriculum reform		
	Collaborative planning, shared vision, and team cohesion		
	Expect involvement and consultation		
	Collaboration among Nursing Education Institutions (NEIs) staff, community, and leaders		
	Involvement of experienced nurse educators in planning processes		
	Alignment with belief systems		
	Stakeholder roles and influence		
	Role clarification and specialization		
	Sense of ownership		
	Collaboration promotes unity and collegiality		
	Poor institutional collaboration		
	Limited engagement from professional bodies		
	Limited stakeholder participation		
	Poor curriculum design and construction		
	Emotional complexity and stress of curriculum development		
5. Faculty capacity development and training needs	Insufficient faculty workshops and capacity‐building sessions	9	[29, 34, 37, 38, 41, 44–46, 48]
	Lack of pedagogical training promotes curriculum implementation confusion		
	Deficient outcomes and lack of teamwork		
	Limited opportunity for faculty development and training		
	Limited continuous workshops for content‐based curriculum (CBC) implementation processes		
	No structured CPD or mentorship		
	Diminished faculty self‐efficacy		
	Inconsistent curriculum implementation measure		

### 2.5. Step 5: Data Presentation

In alignment with Whittemore and Knafl’s [14] assertion that data presentation should be systematically derived from the research focus, the findings of this study are presented through carefully structured tables and figures. This approach ensures clarity, precision, and transparency, thereby enhancing the trustworthiness of the analytical process and facilitating a coherent interpretation of the results [14]. The reviewers reached consensus on the final themes that emerged from the analysis, which are presented as results in the following section.

## 3. Results and Discussion

### 3.1. Results

In this integrative literature review, 22 studies were included in the final analysis. Of these, 2 were nonexperimental quantitative studies, 15 were qualitative studies, and 5 were review papers. The empirical studies were conducted across a range of countries, including the United States of America (*n* = 2), Australia (*n* = 2), the United Kingdom (*n* = 2), New Zealand (*n* = 1), Israel (*n* = 1), China (*n* = 1), Pakistan (*n* = 1), Rwanda (*n* = 2), Namibia (*n* = 1), Lesotho (*n* = 4), and South Africa (*n* = 5). Five themes emerged: leadership influence and change management; lack of quality assurance (QA) in NEIs; inadequate resources to implement a new curriculum; stakeholder engagement, ownership, and collaboration; and faculty capacity development and training needs for curriculum implementation.

#### 3.1.1. Theme 1: Leadership Influence and Change Management

Leadership influence and change management were consistently identified as foundational drivers of curriculum change across 12 studies [28, 30, 32–35, 38, 39, 43, 45, 46, 48]. These studies emphasize that leadership not only sets a strategic vision but also plays a pivotal role in facilitating stakeholder engagement, building institutional readiness, and ensuring continuity throughout the implementation process. For instance, some studies emphasize the importance of distributed and collective leadership in fostering a sense of shared responsibility, which is essential for sustaining curriculum transformation over time [30, 38]. Zhu et al. [19] similarly noted that emotionally intelligent leadership created a supportive environment that enhanced faculty motivation and adaptability. However, a contrasting body of literature [29, 33–35] highlights the pitfalls of inconsistent and hierarchical leadership models. These studies reveal that top‐down decision‐making processes often alienate nurse educators from meaningful participation, undermining their sense of urgency and ownership in the change process. For example, Chowthi–Williams [32] and Brown [29] documented how exclusion from strategic deliberations led to emotional disconnection and a perceived lack of relevance in curricular changes, which in turn subdue innovation and reduce enthusiasm among nurse educators. Furthermore, Ige et al. [34] pointed out that when leadership failed to clarify roles and establish effective communication mechanisms, confusion and resistance multiplied, creating barriers to successful implementation.

Conversely, a more optimistic narrative emerges from studies such as references [29, 43, 46], which highlight the positive outcomes of participatory and well‐structured leadership models. These environments were characterized by institutionalized leadership frameworks, clear role delineations, and supportive management structures. Nyoni and Botma [43] observed that the faculty was more resilient and proactive when leadership demonstrated transparency and accountability, fostering a culture of collaboration. Sodho [46] echoed this by showing that sustained faculty engagement and commitment were higher in institutions where leadership was approachable, inclusive, and invested in ongoing professional development.

Moreover, studies such as references [34, 38] underscore that effective change management requires a coherent and integrated leadership strategy that operates across all levels of the institution. These studies affirm that a clear strategic vision, reinforced by structured support systems, enhances faculty ownership and cultivates an environment conducive to innovation. This aligns with Kotter’s theory of change [50], which emphasizes the need to align leadership behavior with the change vision to maintain momentum and reduce resistance. These studies reveal a nuanced picture of how leadership is undeniably central to curriculum change, and the quality, style, and structure of leadership significantly influence outcomes. Inclusive, transparent, and strategic leadership fosters engagement, strengthens resilience, and drives innovation, whereas top‐down and ambiguous leadership approaches often result in alienation, resistance, and stagnation [50, 51]. This suggests that successful curriculum transformation requires not merely the presence of leadership but leadership that intentionally promotes collaboration, clarity, and sustained support throughout the change process [52, 53].

#### 3.1.2. Theme 2: Lack of Quality Assurance in NEIs

The lack of robust QA mechanisms emerged as a critical impediment to effective curriculum implementation within many NEIs. Eleven (*n* = 11) studies [12, 28, 31, 34, 41–44, 46, 48] consistently reported that institutional (QA) systems were either underdeveloped or misaligned with the objectives of the revised curriculum. Firstly, many NEIs lacked internal monitoring tools capable of systematically tracking the progress and outcomes of curriculum innovation evaluation, and where it occurred, it was often ad hoc, uncoordinated, and inconsistent across departments [12, 31, 43]. This absence of standardized quality indicators and structured feedback mechanisms meant that institutions were unable to assess whether curriculum objectives were being met or whether adjustments were needed [28, 34, 45]. For example, Nyoni and Botma [43] observed that without shared benchmarks, departments developed divergent interpretations of curriculum components, leading to fragmentation and unequal student experience.

Secondly, studies such as references [12, 31, 34, 42, 44, 46] pointed out that weak regulatory oversight and insufficient alignment between institutional QA practices and national standards further compromised transition trustworthiness. While national regulatory bodies may set broad curricular guidelines, their limited involvement in supporting or enforcing QA at the institutional level created a gap between policy and practice. Muraraneza et al. [12] and Sodho et al. [46] both noted that the absence of coordinated external reviews or audits allowed institutional discrepancies to persist unaddressed.

Furthermore, the lack of feedback loop mechanisms through which data from nurse educators could inform iterative improvement meant that lessons from early implementation phases were not systematically captured or acted upon [31, 43, 45]. As highlighted by Baron [29] and supported by Zhu et al. [48], the inability to reflect and respond to implementation challenges undermines the principles of adaptive learning, which are fundamental to curriculum transition and continuous quality improvement (CQI). In contrast, studies proposed in the institutionalization of integrated QA systems are explicitly linked to national nursing education standards and responsive to local institutional contexts [34, 45]. Such systems should include regular internal audits, student and faculty feedback surveys, nurse educators, and performance metrics tied to curriculum goals. Importantly, QA should not be viewed as a compliance exercise but rather as a CQI process that fosters accountability, transparency, and professional growth. Ultimately, cultivating a culture of quality within NEIs requires capacity development in QA literacy, investment in data systems, and leadership that prioritizes evidence‐based practice. When embedded in policy and supported by regular training and institutional incentives, QA mechanisms can serve as the backbone of curriculum transition, ensuring not only implementation fidelity but also long‐term educational excellence.

#### 3.1.3. Theme 3: Inadequate Resources to Implement a New Curriculum

A persistent and significant barrier identified in the reviewed literature was the lack of adequate resources needed to successfully implement a new curriculum. Eleven (*n* = 11) studies from both urban and rural NEIs [12, 28, 30, 31, 37, 41, 44–48] consistently reported a shortage of qualified academic staff, insufficient teaching and learning materials, outdated infrastructure, and limited access to digital and simulation technologies. Human resource shortages were especially concerning in the reviewed literature. Nurse educators in many contexts faced increased workloads, often managing multiple cohorts with limited academic support. Ashipala et al. [28] noted that nurse educators were unable to dedicate enough time to lesson planning or student mentoring, which reduced the depth and quality of curriculum delivery. Similarly, Nyoni and Goddard [44] described that high student‐to‐teacher ratios contributed to faculty burnout and undermined formative assessment and feedback practices.

Muraraneza et al. [12] also highlight the shortage of qualified nurse educators across both academic and clinical settings. The small number of competent nurse educators, combined with the use of inappropriate and outdated teaching methods, hampers the delivery of core curriculum outcomes. This is strongly echoed by Nyoni and Botma [41], who pointed out that nurse educators, even when working within a new curriculum framework, often rely on traditional lecture‐based teaching due to a lack of training, institutional support, or access to modern teaching tools. Repsha et al. [45] identify the burden of managing dual curricula, an issue experienced during the transition period when institutions were required to run both legacy and new curricula concurrently. This dual responsibility was found to strain faculty, dilute focus, and complicate planning and assessment. The transitional workload challenge is validated by references [37, 44, 46], all of whom report significant increases in faculty workload, administrative pressure, and emotional fatigue. Similarly, Zhu et al. [48] highlight the emotional toll and institutional pressure faced by nurse educators during curriculum transition. Heavy teaching loads, unrealistic performance expectations, and limited support systems contribute to burnout and disengagement.

Furthermore, the absence of suitable textbooks and teaching models hampers nurse educators’ ability to turn theory into practice, affecting the curriculum’s effectiveness and coherence. Zhu et al. [48] study is notable for addressing psychological resilience, international exposure, and practical teaching strategies, while also acknowledging common obstacles such as student resistance, high workloads, and institutional demands. Botlhoko et al. [31] offer a unique perspective by emphasizing transportation issues for clinical supervision as a key, context‐specific logistical challenge. This detail highlights the operational realities of rural and underresourced institutions where reaching clinical sites is vital for hands‐on training but remains logically difficult. These cumulative challenges reinforce the need for a systemic, well‐resourced approach to curriculum change that prioritizes faculty support, sustainable workloads, and infrastructure development to ensure effective implementation and long‐term success.

#### 3.1.4. Theme 4: Stakeholder Engagement, Ownership, and Collaboration

Stakeholder engagement emerged as a pivotal theme in curriculum transition, emphasizing the necessity for inclusive, transparent, and participatory processes. Effective curriculum change requires the involvement of a broad spectrum of stakeholders, including nurse educators, students, preceptors, professional councils, local partners, and community representatives, to ensure transition is contextually grounded, responsive, and sustainable. Numerous (*n* = 11) studies [28–31, 33, 34, 36, 40, 44, 47, 48] reported the predominance of top‐down approaches in curriculum development processes. In these contexts, key stakeholders, nurse educators, were often marginalized or engaged superficially. This limited participation led to a lack of shared understanding and ownership, resulting in weak alignment between curriculum goals and the realities of practice settings. For example, Ige et al. [39] highlighted how exclusion from early planning stages contributed to nurse educator’s resistance, confusion about curriculum structure, and limited preparedness to implement change.

In contrast, participatory models, as described in references [34, 48], demonstrate the benefits of early and continuous stakeholder engagement. These models included mechanisms such as curriculum planning committees, consultative workshops, and feedback loops that allowed for the co‐creation of curriculum content. Institutions that fostered interprofessional collaboration, encouraged open dialog, and promoted transparency in decision‐making were better positioned to build trust, align expectations, and develop curricula that reflected local needs and global best practices. Zhu et al. [48] further emphasized that such inclusive approaches fostered a culture of collective responsibility and resilience during curriculum implementation.

Failure to engage stakeholders meaningfully created a sense of disconnect, reinforcing hierarchical dynamics and perpetuating resistance to change [43]. Nurse educators and students who felt excluded were less likely to engage with the transition process. At the same time, clinical partners, who play a vital role in bridging theory and practice, often found themselves ill‐prepared to support the new curriculum due to their lack of involvement in curriculum planning. As the literature illustrates, true collaboration is characterized by shared decision‐making, mutual respect, and joint accountability [33, 48]. Stakeholder inclusion enhances the legitimacy and acceptability of curriculum transition, also ensuring its contextual relevance varies widely [34].

From a broader perspective, the stakeholder collaborative is increasingly acknowledged in global policy frameworks such as the UNESCO and UNESCO’s education for sustainable development goals [54]. A curriculum transition that prioritizes stakeholder engagement, fosters ownership, and develops collaboration networks is more likely to succeed. Such approaches not only enhance implementation readiness but also promote innovation, contextually relevant, and long‐term sustainability.

#### 3.1.5. Theme 5: Faculty Capacity Development and Training Needs

Faculty preparation emerged as a critical theme across 9 of the 22 review studies, underscoring the central role of nurse educators in transitioning curriculum change into meaningful classroom and clinical practice, Despite being tasked with implementing complex, outcome‐based, and competency‐based curricula, many nurse educators lacked the necessary pedagogical training, curriculum literacy, and practical support to navigate this shift effectively [29, 34, 38, 41, 42, 44–46, 48]. In addition, the transition to CBE necessitates more than familiarity with content; it demands a paradigm shift in teaching philosophy, assessment strategies, and learner‐centered methodologies. Yet, studies revealed that many nurse educators were insufficiently prepared to adopt these changes. Also, nurse educators often continue to use traditional, didactic approaches due to a lack of exposure to interactive, reflective, and practice‐oriented pedagogies that underpin CBE [44, 45].

Best practices were highlighted in studies such as Zhu et al. [48], where structured peer mentorship, continuous professional development, and international faculty exchange programs significantly improved nurse educators confidence and competence. These initiatives provided platforms for knowledge‐sharing, professional reflection, and collaborative problem‐solving which are essential for cultivating a responsive and adaptive teaching workforce. Similarly, studies emphasized that tailored CPD programs focusing on curriculum implementation, learner assessment, and digital teaching tools were linked to improved educator engagement and student outcomes [38, 44]. However, in many institutions, faculty development was approached superficially, delivered through intermittent workshops, short‐term seminars, or self‐directed learning modules that failed to address the diverse learning needs, experiences, and disciplinary backgrounds of academic staff [29, 42, 45]. Baron [29] criticized these approaches as reactive and fragmented, often lacking follow‐up, contextual relevance, or alignment with institutional transition goals.

The findings affirm that curriculum change requires more than structural or policy‐level adjustments; it necessitates transformative, long‐term capacity development that is strategic, contextualized, and embedded within institutional reform frameworks [37, 38, 44]. Effective faculty development should be ongoing, scaffolded, and inclusive, offering differentiated learning pathways that support novice and experienced nurse educators alike [29, 34]. Furthermore, development initiatives must address not only pedagogical skills but also change leadership, emotional resilience, and technological integration, which are critical in rapidly evolving educational environments [55]. This includes allocating dedicated budgets for CPD, establishing mentoring networks, and linking faculty training to career progression and institutional recognition [38, 48]. Without a committed investment in faculty capacity development, curriculum shifts risk stagnation at the implementation stage [45]. A well‐supported, pedagogically confident, and collaborative academic workforce is essential for curriculum success and for fostering a culture of innovation and continuous improvement in nursing education [56, 57].

## 4. Discussion

Public and private institutions differ structurally, culturally, and strategically, and these differences significantly influence leadership approaches, governance models, and change management processes [53, 56, 57]. Public institutions operate within bureaucratic, policy‐driven frameworks, often characterized by multiple layers of accountability to the government, regulatory bodies, and the public [58]. Decision‐making tends to be slower due to compliance requirements, union agreements, and public funding oversight. In contrast, private institutions generally have greater managerial autonomy, allowing for more flexible governance structures and faster strategic responses [55].

Leadership in public institutions often emphasizes procedural fairness, stakeholder engagement, and transparency, reflecting democratic governance principles [57]. However, rigid hierarchies may limit innovation and slow responsiveness. Private institutions, by contrast, may adopt more entrepreneurial and performance‐driven leadership models, emphasizing efficiency, market competitiveness, and innovation [59]. While this can promote agility, it may also risk prioritizing financial outcomes over collaborative engagement. Public institutions rely heavily on government funding, which may be constrained and politically influenced [60]. Budget allocations are often fixed within fiscal cycles, limiting rapid reallocation for innovation. Private institutions depend more on tuition revenue, private investment, or endowments, leading to stronger market responsiveness and also financial vulnerability if enrollments decline [61]. In nursing education, private institutions may more quickly invest in simulation technology, whereas public institutions may require extended procurement processes. Research suggests that inclusive and participatory leadership enhances employee commitment in both sectors, but public‐sector change often requires more extensive stakeholder consultation [62].

The objective of this integrative literature review was achieved as the best available evidence for nurse educators on transitioning from one curriculum to another in NEIs. The five themes that emerged were: leadership influence and change management; lack of QA in nursing education; inadequate resources to implement a new curriculum; stakeholder engagement, ownership, and collaboration; and faculty development and training needs.

The first theme, *Leadership influence and change management*, highlights that effective leadership and well‐structured change management are central to successful transitions in NEIs. Some studies consistently highlight the critical role of leadership in setting a clear strategic vision, fostering stakeholder engagement and building institutional readiness for change. For example, some studies emphasize the value of distributed and emotionally intelligent leadership styles, which promote shared responsibility and faculty adaptability [26, 35, 48]. These inclusive leadership models create supportive environments that facilitate collaboration and continuity throughout the change process, aligning with Kotter’s model of change [50] that stresses the importance of empowering others and creating short‐term wins to sustain momentum. These findings affirm that for nurse educators to transition from one curriculum to another successfully, institutional leadership must embrace coherent and inclusive change management strategies that foster a culture of trust, collaboration, and CPD.

The second theme, *Lack of quality assurance in nursing education institutions,* emphasizes that the absence of strong, structured, and coordinated QA systems creates a major obstacle to effective curriculum transition in NEIs. The absence of robust, structured, and coordinated QA systems compromises the ability of NEIs to consistently monitor, evaluate, and improve educational processes, teaching methodologies, and clinical training. Weak QA mechanisms result in variability in graduate competence, inconsistencies in curriculum implementation, and limited capacity to respond to evolving health system needs [8, 60]. This challenge is particularly evident in both resource‐limited and rapidly expanding higher education contexts, where NEIs struggle to align program outcomes with national and international standards, maintain accreditation, and ensure institutional preparedness.

Strengthening QA frameworks is therefore essential to safeguard the quality of nursing education, as well as to support sustainable curriculum transitions, foster innovation, and enhance workforce readiness to meet complex and diverse healthcare demands [5, 9]. In the absence of systematic and integrated internal and external QA processes, curriculum reforms risk remaining superficial and policy‐driven rather than becoming embedded and sustainable transformations [8, 9]. Effective QA should function as a dynamic governance mechanism that balances innovation with academic rigor; institutions with strong QA cultures are better positioned to monitor implementation, address development gaps, and adapt curricula, whereas weak systems undermine reform sustainability [5, 60].

The third theme, *Inadequate resources to implement a new curriculum*, aligns with broader research indicating that adequate resourcing is a global challenge in nursing education. According to WHO [23], the global shortage of nurse educators and infrastructure gaps significantly compromise training and health system preparedness. Similarly, studies emphasize that without sufficient faculty development, institutional investment, and modernized learning environments, educational shift efforts are likely to weaken [55, 63]. The findings reinforce the urgent need for strategic, system‐wide investment in human, physical, and technological resources to support a sustainable and effective transition to changed nursing curricula that meet the demands of contemporary healthcare systems. The WHO [23] states that fair and sufficient investment in education infrastructure, technology, and workforce development is essential to transforming the health education system. Without this investment, curriculum transitions, no matter how well planned, risk failure due to structural issues rather than teaching flaws.

The fourth theme, *Stakeholder engagement, ownership, and collaboration,* highlights that stakeholder engagement is a crucial factor in the success of curriculum transition in NEIs. Effective transitioning relies on strong curriculum design and the meaningful involvement of diverse stakeholders such as nurse educators, students, clinical practitioners, regulatory bodies, relevant partners, and community members [29, 33, 34]. These groups offer valuable perspectives that help ensure relevance and feasibility. Factors such as role clarification ensure a clear delineation of responsibilities among key stakeholders (nurse educators, clinical preceptors, curriculum developers, regulatory bodies, and students), thereby enabling students to understand how curriculum decisions are made and how theory is translated into clinical practice [34, 38]. Specialization complements this by ensuring that curriculum development is guided by experts in pedagogy, clinical practice, assessment, and policy, allowing students to appreciate the complexity and standards underpinning curriculum design [45]. Role clarification and specialization elements promote a coherent and responsive curriculum, enhance student learning outcomes, and support meaningful student participation in feedback and evaluation processes, ultimately strengthening their professional identity and ensuring alignment with evolving healthcare demands [1, 43, 48]. Inclusive and participatory methods like collaborative curriculum design, ongoing feedback, and stakeholder‐led decision‐making build trust, foster a shared vision, and enhance institutional readiness for change. Global frameworks such as the WHO’s Global Strategic Directions for Nursing and Midwifery (2021–2025) and the Global Health Workforce Network advocate for stakeholder co‐creation as essential for work‐responsive education [1]. Likewise, UNESCO [64] stresses that participatory governance boosts innovation, improves local adaptability, and ensures long‐term sustainability in nursing systems. By embedding stakeholder collaboration into every phase of curriculum development and implementation, NEIs can better manage complex transitions, promote equitable outcomes, and prepare nursing graduates who are capable, confident, and aware of their context.

The fifth theme, *Faculty capacity development and training needs,* the best available evidence from this integrative literature review indicates that effective curriculum transition in NEIs depends on comprehensive institutional preparation. Studies show many nurse educators lack the pedagogical expertise, curriculum literacy, and institutional support needed to implement competency‐based and learner‐centered curricula [38, 44, 48, 65]. Successful transitions are linked to ongoing, context‐sensitive professional development, including peer mentorship and embedding training into institutional change frameworks [1]. Therefore, to achieve meaningful and sustainable curriculum transitioning, faculty development must be strategic, ongoing, and institutionalized, supporting educators through tailored training, QA integration, and structured incentives. Faculty capacity development is essential for successful and sustainable curriculum transition in nursing education. Ongoing professional development encompassing pedagogical skills, assessment literacy, curriculum design, clinical facilitation, leadership, and change management is necessary to support shifts toward nursing education and to ensure effective, long‐term implementation [5, 8, 10, 51].

## 5. Strengths, Limitations, and Implications

### 5.1. Strengths

This integrative literature review draws on a broad and diverse range of literature, offering a comprehensive analysis of both structural and relational factors influencing curriculum transitioning in NEIs. It emphasizes the vital role of transformative leadership, particularly distributed and emotionally intelligent approaches, in enhancing institutional readiness and faculty engagement [38]. The review also emphasizes the importance of stakeholder participation in ensuring transition legitimacy and contextual relevance, as participatory models foster ownership and innovation [46, 48]. Furthermore, it emphasizes effective faculty development initiatives, such as structured CPD and international exchanges, as crucial enablers of CBE. By addressing systemic challenges, including managing dual curricula and weak QA mechanisms, the review goes beyond surface‐level analysis to reveal deeper barriers to successful transition to a new curriculum.

### 5.2. Limitations

Although the inclusion criteria were broadened to capture public, private, and mixed higher education contexts, the transferability of findings specifically to NEIs may be influenced by contextual differences in governance, regulation, and resource availability. There is a lack of long‐term research on the effects of leadership, QA, and faculty development on transitioning within NEIs. Student perspectives are notably unrepresented, despite their key roles in curriculum outcomes. In addition, QA mechanisms are often fragmented and not aligned with national standards, and faculty development efforts tend to be inconsistent, lacking relevance to context and strategic planning.

### 5.3. Implications

The findings carry implications for policy, institutional planning, and research. Leadership development should be integrated into transition strategies, promoting inclusive, emotionally intelligent, and distributed leadership at all levels. QA needs to shift from a compliance‐based approach to comprehensive, ongoing improvement systems aligned with national standards. Addressing persistent resource constraints, especially in rural NEIs, is essential for equitable curriculum delivery. Faculty development should be continuous, strategically aligned, and tailored to diverse needs, with investments in mentorship, pedagogy, and digital skills. Finally, stakeholder engagement must be regarded as a strategic priority, ensuring meaningful collaboration among all actors to enhance curriculum relevance, responsiveness, and sustainability.

## 6. Conclusion

This review underscores that curriculum transitioning in NEIs is a complex, multidimensional process that extends beyond content revision. It requires the coordinated alignment of leadership, resources, stakeholder engagement, QA, and faculty development factors that are especially critical in NEIs facing systemic and historical challenges. The evidence highlights that the most impactful curriculum transitions are those led by visionary, participatory leadership that cultivates ownership among stakeholders and invests in continuous professional development. Conversely, top‐down rigidly imposed change efforts that often lack transparent communication and stakeholder involvement frequently result in resistance, confusion, and implementation fatigue.

Robust institutional support structures, collaborative decision‐making platforms, and embedded QA systems emerge as indispensable conditions for meaningful and lasting curricular transformation. Ultimately, the review affirms that curriculum transitioning cannot be effective without long‐term strategic commitment and a whole‐system approach. Institutional readiness, adequate resourcing, and a shared vision for educational excellence are vital to achieving transformative outcomes. Unless NEIs proactively align governance, infrastructure, and human capital with transition goals, they risk falling short of preparing competent, adaptable nursing graduates capable of meeting the evolving demands of 21st‐century healthcare environments.

## Author Contributions

Nomawabo Lessie Luzipo was responsible for conceptualization, methodology, conducting the literature review, and preparing the original manuscript draft. Khumoetsile Daphney Shopo and Richelle Van Waltsleven provided supervision and critical input through review and editing.

## Funding

The authors received no financial support for the research, authorship, and/or publication of this article.

## Disclosure

All authors read and approved the final version of the manuscript.

## Conflicts of Interest

The authors declare no conflicts of interest.

## Data Availability

The data that support the findings of this study are available from the corresponding author upon reasonable request.

## References

[1] World Health Organization , Global Strategic Directions for Nursing and Midwifery, Geneva: WHO, 2021, https://iris.who.int/bitstream/handle/10665/344562/9789240033863eng.pdf?sequence=1.

[2] Phillips J. , Vinten S. A. , and Koshy K. , Curriculum Innovation in Nurse Education: A Literature Review, Nurse Education Today. (2013) 33, no. 1, 91–95.

[3] Awadhalla M. , Al-Mohandis B. , and Al-Darazi F. , Transformation of Nursing Education: The Experience of Bahrain, Eastern Mediterranean Health Journal. (2018) 24, no. 9, 959–964, 10.26719/2018.24.9.959, 2-s2.0-85058377349. 30570129

[4] Solheim K. , Leners N. , and Pape K. , Sustaining a Concept-Based Curriculum: Beyond the Launch, Journal of Professional Nursing. (2022) 38, no. 4, 239–246.

[5] Giddens J. F. , Concept-Based Curriculum and Instruction for the Thinking Classroom, 2017, 2nd edition, Elsevier, St. Louis.

[6] Iwasiw C. L. and Goldenberg D. , Curriculum Development in Nursing Education, 2020, 4th edition, Jones & Bartlett Learning, Burlington, MA.

[7] Johnson M. and Cowin L. S. , Measuring the Qualities of Nurses: Development and Testing of the Qualities of Nurses Scale, Nursing Education Perspectives. (2013) 34, no. 2, 111–117, 10.1097/00024776-201303000-00009. 23763025

[8] World Health Organization , Nurse Educator Core Competencies, 2016, WHO, Geneva.

[9] International Council of Nurses , Guidelines on Advanced Practice Nursing, 2021, ICN, Geneva.

[10] Billings D. M. and Halstead J. A. , Teaching in Nursing: A Guide for Faculty, 2023, 7th edition, Elsevier, St. Louis.

[11] Darbyshire P. and McKenna L. , Nursing Curriculum Reform: a Time for Innovation, Nurse Education Today. (2021) 104, no. 1, 1–8.

[12] Muraraneza C. , Mtshali N. G. , and Mukamana D. , Issues and Challenges of Curriculum Reform to Competency-based Curricula in Africa: A Meta-Synthesis, Nursing and Health Sciences. (2017) 19, no. 1, 5–12, 10.1111/nhs.12316, 2-s2.0-84997522621. 27805792

[13] Gupta S. , Narayanasamy M. , Hwang S. , and Patel K. , Strategies for Fostering Resilience and Adaptability in Nurse Educators, Journal of Professional Nursing. (2023) 46, no. 1, 45–51.37188421

[14] Whittemore R. and Knafl K. , The Integrative Review: Updated Methodology, Journal of Advanced Nursing. (2005) 52, no. 5, 546–553, 10.1111/j.1365-2648.2005.03621.x, 2-s2.0-28044463360. 16268861

[15] Win K. C. M. , Zhou H. , Patton V. , Steen M. , and Della P. , Factors Contributing to Non-Adherence to Treatment Among Adult Patients With Long-Term Haemodialysis: An Integrative Review, Nursing Reports. (2025) 15, no. 9, 1–43, 10.3390/nursrep15090314. PMC12472532 41003269

[16] Ayobi Z. , Christopher C. , and Naidoo D. , Caregiver’s Perceptions of Their Role in Early Childhood Development and Stimulation Programmes in the Early Childhood Development Phase Within a Sub-Saharan African Context: an Integrative Review, South African Journal of Occupational Therapy. (2021) 51, no. 3, 84–92, 10.17159/2310-3833/2021/vol51n3a10.

[17] Woo M. W. J. and Avery M. J. , Nurses’ Experiences of Involuntary Error Report: an Integrative Literature Review, International Journal of Nursing Science. (2021) 8, no. 1, 453–469, 10.1016/j.ijnss.2021.07.004. PMC8488811 34631996

[18] Lima M. S. and Alzyood M. , The Impact of Preceptorship on the Newly Qualified Nurse and Preceptors Working in a Critical Care Environment: An Integrative Literature Review, 2024, https://onlinelibrary.wiley.com/doi/pdf/10.1111/nicc.13061. 10.1111/nicc.13061 38511618

[19] Kurup C. , Betihavas V. , Burston A. , and Jacob E. , Strategies Employed by Developed Countries to Facilitate the Transition of Internationally Qualified Nurses’ Specialty Skills into Clinical Practice*:* An Integrative Review, Nursing Open. (2023) 10, no. 12, 7528–7543, 10.1002/nop2.2023. 37794722 PMC10643820

[20] Zenani N. E. , Sehularo L. A. , Gause G. , and Chukwuere P. C. , The Contribution of Inter-Professional Competent Undergraduate Nursing Students: An Integrative Literature Review, BMC Nursing Journal. (2023) 22, no. 315, 1–12. 10.1186/s12912-023-01482-8 PMC10500801 37710257

[21] Moher D. , Liberati A. , Tetzlaff J. , and Altman D. G. , Preferred Reporting Items for Systematic Reviews and Meta-Analyses: The PRISMA Statement, PLoS Medicine. (2009) 6, no. 7, 10.1371/journal.pmed.1000097, 2-s2.0-68049122102. PMC2707599 19621072

[22] Dhollande S. , Sharma K. , Krishnan M. , and Zahir S. , Use of Critical Appraisal Tools in Evidence Synthesis: A Review, International Journal of Evidence-Based Healthcare. (2021) 19, no. 4, 427–434.

[23] World Health Organisation , State of the World’s Nursing 2020: Investing in Education, Jobs and Leadership, 2020, World Health Organization, Geneva.

[24] Polit F. D. and Beck C. T. , Essentials of Nursing Research: Appraising Evidence for Nursing Practice, 2021, 11th edition, Kluwer.

[25] Page M. J. , McKenzie J. E. , Bossuyt P. M. et al., The PRISMA 2020 Statement: An Updated Guideline for Reporting Systematic Reviews, BMJ. (2021) 1, no. 372, 1–71, 10.1136/bmj.n71. PMC8005924 33782057

[26] Covidence , Covidence systematic review software [Computer program], 2025, Veritas Health Innovation, Melbourne, Australia, https://www.covidence.org.

[27] Shopo K. D. , Nuuyama V. , and Chihururu L. , Enhancing Cultural Competence in Undergraduate Nursing Students: An Integrative Literature Review Strategy for Institutions of Higher Education, Journal of Transcultural Nursing. (2024) 36, no. 4, 412–428. 39648427 10.1177/10436596241301407 PMC12179409

[28] Ashipala D. O. , Likando G. , and Ndumbu S. , Facilitators and Barriers to Nursing Educators in Implementing an Undergraduate Nursing and Midwifery Curriculum in the Digital Era, IGI Global. (2024) 1, no. 1, 21–44.

[29] Baron k. A. , Changing to Concept-Based Curricula: The Process for Nurse Educators, The Open Nursing Journal. (2017) 11, no. 1, 277–287, 10.2174/1874434601711010277, 2-s2.0-85043759412. 29399236 PMC5759089

[30] Molise N. A. , Botma Y. , and Van Jaarsveldt D. , A Socially Constructed Framework for Culturally Congruent Nursing Curriculum Transformation in Lesotho: A Multi-Methods Approach, International Journal of Africa Nursing Sciences. (2024) 21, no. 1, 1–7.

[31] Botlhoko K. P. , Zenani N. E. , and Sehularo L. A. , Experiences of Nurse Educators Regarding the R171 Nursing Curriculum in North-West Province, South Africa, Sage Open Nursing. (2024) 10, no. 1, 1–9, 10.1177/23779608241293700. PMC11638997 39676902

[32] [Chowthi-Williams A. , Evaluation of How a Real-Time Pre-Registration Health Care Curricula was Managed Through the Application of a Newly Designed Change Management Model: A Qualitative Case Study, Nurse Education Today. (2018) 61, no. 1, 242–248, 10.1016/j.nedt.2017.12.004, 2-s2.0-85038376537. 29272823

[33] Chowthi-Williams A. , Joan C. , and Stephen L. , Evaluation of How a Curriculum Change in Nurse Education was Managed Through the Application of a Business Change Management Model: A Qualitative Case Study, Nurse Education Today. (2016) 36, no. 1, 133–138, 10.1016/j.nedt.2015.08.023, 2-s2.0-85027919682. 26372610

[34] Ige W. B. , Ngcobo W. B. , and Afolabi O. , Implementation of Competency-Based Education for Quality Midwifery Programmes in Africa: A Scoping Review, BMC Nursing. (2024) 23, no. 1, 10.1186/s12912-024-02333-w. PMC11438267 39334130

[35] Kemelman R. and Cojocaru D. , Nursing Educators’ Concerns During the Implementation of a Core Curriculum, Kontakt. (2024) 26, no. 3, 238–245, 10.32725/kont.2024.033.

[36] Keogh J. J. , Fourie W. J. , Watson S. , and Gay H. , Involving the Stakeholders in the Curriculum Process: A Recipe for Success, Nurse Education Today. (2010) 30, no. 1, 37–43, 10.1016/j.nedt.2009.05.017, 2-s2.0-71349086177. 19560237

[37] Martin S. , Richards C. , Keogh S. , and Ward A. , Embedding Planetary Health in Nursing Education: Exploring the Barriers and Enablers to Implementing Changes in Undergraduate Bachelor of Nursing Curriculum, Teaching and Learning in Nursing. (2024) 19, no. 2, 261–268, 10.1016/j.teln.2023.11.008.

[38] Meyer A. E. and Olsen J. M. , Engaging Clinical Partners in Curricular Initiatives to Improve Practice Readiness, Journal of Nursing Education. (2023) 62, no. 12, 706–710, 10.3928/01484834-20231006-08. 38049307

[39] Molise N. A. , Botma Y. , and Van Jaarsveldt D. , Cultural Barriers and Facilitators for Transformative Nursing Curriculum Transformation, International Journal of Africa Nursing Sciences. (2023) 18, no. 1, 1–6, 10.1016/j.ijans.2023.100550.

[40] Muraraneza C. and Mtshali G. N. , Planning Reform to Competency-based Curricula in Undergraduate Nursing and Midwifery Education: A Qualitative Study, Nurse Education Today. (2021) 106, no. 1, 1–6, 10.1016/j.nedt.2021.105066. 34340195

[41] Nyoni C. N. and Botma Y. , Sustaining a Newly Implemented Competence-based Midwifery Programme in Lesotho: Emerging Issues, Midwifery. (2018) 62, no. 1, 105–117. 10.1016/j.midw.2018.01.015 29421640

[42] Nyoni C. N. and Botma Y. , Implementing a Competency-based Midwifery Programme in Lesotho: a Gap Analysis, Nurse Education in Practice. (2019) 34, no. 1, 72–78, 10.1016/j.nepr.2018.11.005, 2-s2.0-85057176859. 30466039

[43] Nyoni C. N. and Botma Y. , Integrative Review on Sustaining Curriculum Change in Higher Education: Implications for Nursing Education in Africa, International Journal of Africa Nursing Sciences. (2020) 12, no. 1, 1–8, 10.1016/j.ijans.2020.100208.

[44] Nyoni C. N. and Goddard V. C. T. , Needs of Early Adopters in Supporting a Nursing Curriculum Innovation in a low-resource Setting: An Exploratory Case Study, Nurse Education Today. (2021) 104, no. 1, 1–6, 10.1016/j.nedt.2021.105002. 34126325

[45] Repsha C. L. , Quinn B. L. , and Peters A. B. , Implementing a Concept-Based Nursing Curriculum: A Review of the Literature, Teaching and Learning in Nursing. (2020) 15, no. 1, 66–71, 10.1016/j.teln.2019.09.006.

[46] Sodho M. S. , Fatima M. , and Abbas Z. , Challenges Faced by Nursing Faculty in Curriculum Implementation in Nursing Schools of Sindh: Nurses Faculty Perspectives, Journal of the Liaquat University of Medical and Health Sciences. (2021) 20, no. 5, 363–369.

[47] Woods A. , Exploring Unplanned Curriculum Drift, Journal of Nursing Education. (2015) 54, no. 11, 641–644, 10.3928/01484834-20151016-05, 2-s2.0-84946910423. 26517076

[48] Zhu Y. , Pei X. , and Chen X. , Faculty’s Experience in Developing and Implementing Concept-based Teaching of Baccalaureate Nursing Education in the Chinese Context: A Descriptive Qualitative Research Study, Nurse Education Today. (2022) 108, no. 1, 1–6, 10.1016/j.nedt.2021.105126. 34601151

[49] Critical Appraisal Skills Programme) , CASP Checklists, 2018, https://casp-uk.net/casp-tools-checklists/.

[50] Kotter J. P. , Change: How Organisations Achieve hard-to-imagine Results in Uncertain and Volatile Times, 1996, New Jersey: Wiley & Sons.

[51] Matahela T. and van Rensburg G. H. , Enhancing Nurse Faculty Resilience Through self-leadership: Guidelines for Resource Mobilization in Dynamic Academic Environments, Frontiers in Psychology. (2024) 15, 10.3389/fpsyg.2024.1280561. PMC11554518 39534479

[52] Appelbaum S. H. , Habashy S. , Malo J. L. , and Shafiq H. , Back to the Future: Revisiting Kotter’s 1996 Change Model, Journal of Management Development. (2021) 40, no. 1, 5–17, 10.1108/JMD-05-2020-0144.

[53] Todnem By R. , Organizational Change Management: A Critical Review, Journal of Change Management. (2021) 21, no. 1, 33–61, 10.1080/14697017.2020.1850731.

[54] Unesco , Global Education Monitoring Report 2020: Inclusion and Education–all Means all, 2020, UNESCO, Paris.

[55] World Health Organization , Global Report on Health Education Transformation, 2023, World Health Organization, Geneva, https://iris.who.int/bitstream/handle/10665/93635/9789241506502_eng.pdf.

[56] Boyne G. A. , Public and Private Management: What’s the Difference?, Journal of Public Administration Research and Theory. (2021) 31, no. 2, 330–347.

[57] Van Wart M. , Roman A. , Wang X. , and Liu C. , Operationalizing the Definition of Public Leadership, The American Review of Public Administration. (2021) 51, no. 6, 1–15.

[58] Christensen T. , Laegreid P. , and Rykkja L. H. , Organizing for Crisis Management: Building Governance Capacity and Legitimacy, Public Administration Review. (2020) 80, no. 5, 774–785. 32836445 10.1111/puar.13241 PMC7280699

[59] Anderson D. and Anderson L. A. , Beyond Change Management: How to Achieve Breakthrough Results Through Conscious Change Leadership, 2020, 2nd edition, Wiley.

[60] Oecd , Education at a Glance 2021: OECD Indicators, 2021, OECD Publishing, Paris.

[61] Marginson S. , High Participation Systems of Higher Education, Oxford Review of Education. (2022) 48, no. 2, 1–19.

[62] Vakola M. , Tsaousis I. , and Nikolaou I. , Change Recipients’ Reactions to Organizational Change: a meta-analytic Review, Journal of Change Management. (2021) 21, no. 3, 1–31.

[63] Campbell J. , Dussault G. , Buchan J. et al., A Universal Truth: No Health Without a Workforce, 2021, World Health Organization, Geneva.

[64] Unesco , Global Education Monitoring Report 2021/2: Non-State Actors in Education, 2021, UNESCO, Paris.

[65] Frenk J. , Chen L. , Bhutta Z. A. et al., Health Professionals for a New Century: Transforming Education to Strengthen Health Systems in an Interdependent World, The Lancet Commissions. (2010) 376, no. 1, 1923–1958, 10.1016/s0140-6736(10)61854-5, 2-s2.0-78649871039. 21112623

